# Genetic Testing Goes Beyond Imaging and Histological Evaluation in Pleuroparenchymal Fibroelastosis

**DOI:** 10.1007/s00408-024-00685-3

**Published:** 2024-03-10

**Authors:** Ghadah Alrehaili, Jennifer Kemppainen, Sanjay Kalra, Filippo Pinto e Vairo, Teng Moua, Eunhee S. Yi, Alejandro Ferrer, Mrinal M. Patnaik, Eva M. Carmona

**Affiliations:** 1 Department of Pulmonary and Critical Care, Mayo Clinic, Rochester, MN USA; 2 Center for Individualized Medicine, Mayo Clinic, Rochester, MN USA; 3 Clinical Genomics, Mayo Clinic, Rochester, MN USA; 4 Department of Laboratory Medicine and Pathology, Mayo Clinic, Rochester, MN USA; 5 Hematology/Oncology Departments, Mayo Clinic, Rochester, MN USA

**Keywords:** Telomere biology disorders, Surfactant protein disorders, SFTPA2, Pleuroparenchymal fibroelastosis (PPFE), Lung genetics, Pulmonary fibrosis

## Abstract

**Background:**

Lung biopsy remains the gold standard in the diagnosis of fibrotic interstitial lung disease (F-ILD), but there is a growing appreciation of the role of pathogenic gene variants in telomere and surfactant protein genes, especially in familial pulmonary fibrosis (FPF). Pleuroparenchymal fibroelastosis (PPFE) is a rare disease that can coexist with different patterns of F-ILD, including FPF. It can be progressive and often leads to respiratory failure and death. This study tested the hypothesis that genetic testing goes beyond radiological and histological findings in PPFE and other F-ILD further informing clinical decision-making for patients and affected family members by identifying pathological gene variants in telomere and surfactant protein genes.

**Methods:**

This is a retrospective review of 70 patients with F-ILD in the setting of FPF or premature lung fibrosis. Six out of 70 patients were diagnosed with PPFE based on radiological or histological characteristics. All patients underwent telomere length evaluation in peripheral blood by Flow-FISH or genetic testing using a customized exome-based panel that included telomere and surfactant protein genes associated with lung fibrosis.

**Results:**

Herein, we identified six individuals where radiographic or histopathological analyses of PPFE were linked with telomere biology disorders (TBD) or variants in surfactant protein genes. Each case involved individuals with either personal early-onset lung fibrosis or a family history of the disease. Assessments of telomere length and genetic testing offered insights beyond traditional radiological and histopathological evaluations.

**Conclusion:**

Detecting anomalies in TBD-related or surfactant protein genes can significantly refine the diagnosis and treatment strategies for individuals with PPFE and other F-ILD.

## Introduction

Lung biopsy is considered the gold standard method for diagnosing fibrotic interstitial lung disease (F-ILD). There is, however, a growing appreciation of the role of germline variants in telomere-related and surfactant protein-related genes, especially in those with familial pulmonary fibrosis (FPF) [1, 2]. There are no pathognomonic radiological or histological findings suggestive of an underlying genetic disorder in F-ILD. In fact, relatives with familial pathogenic genetic variants can present with different radiological and histological findings [3, 4]. Conversely, patients with similar lung histology may have different underlying genetic disorders. Identifying pathogenic variants is important as it may further inform clinical decision-making and avoid invasive procedures like lung biopsy. Herein, we present 6 cases of pleuroparenchymal fibroelastosis (PPFE) and one case with usual interstitial pneumonia (UIP) for whom genetic testing added to the radiographic and histological findings.

## Methods

This is a retrospective review of patients presenting to the interstitial lung disease (ILD) clinic for evaluation of F-ILD in the setting of FPF or premature lung fibrosis between January 2016 and December 2023. Out of the 70 patients evaluated with telomere length and genetic assessment, 6 were diagnosed PPFE. A diagnosis of familial disease necessitated the presence of at least two affected family members within three generations, with premature lung fibrosis defined as onset before the age of 50 [1]. Criteria for PPFE included either radiological or histological confirmation. Definitive PPFE histopathology was defined as the involvement of upper pleura and lung parenchyma with the presence of fibrosis and elastosis, while radiological imaging was defined as upper lung field fibrotic changes along with thickening of the pleura [5, 6]. Radiological and histological UIP was defined as per ATS guidelines [7]. Telomere length in peripheral blood was determined clinically by Flow-FISH (Johns Hopkins University Laboratory, Baltimore, MD). Genetic testing was performed using DNA extracted from whole blood using a customized exome-based panel through a CLIA-certified laboratory that included the following genes: *ABCA3, ACD, CSF2RA, CSF2RB, CTC1, DKC1, ELMOD2, FLCN, GBA, HPS1, HPS4, NAF1, NF1, NHP2, NOP10, PARN, RTEL1, SFTPA1, SFTPA2, SFTPB, SFTPC, SLC34A2, SLC7A7, SMPD1, STAT3, TERC, TERT, TINF2, TMEM173, TSC1, TSC2, USB1, WRAP53*. Genetic variants were reported according to the American College of Medical Genetics (pathogenic, likely pathogenic, variant of unknown significance, likely benign or benign) [8]; clinical data was extracted from the electronic medical records. This study was approved by the local IRB (#23-001833).

## Results

Among patients evaluated for familial pulmonary fibrosis (FPF) or early-onset pulmonary fibrosis, six cases (6/70–8.6%) were identified with PPFE The demographic and clinical characteristics of these patients reveal noteworthy differences between those with PPFE and the remaining cohort as shown in Table 1 A significant gender disparity was observed, with the PPFE group predominantly female (*n* = 5) compared to the majority male composition of the non-PPFE group (*n* = 23), *p* = 0.0235. Pulmonary function parameters reveal a significantly lower mean forced vital capacity (FVC) % predicted for the PPFE group (47.8, SD ± 19.3) compared with the non-PPFE group (74.8. SD ± 21.0), *p* = 0.017. No statistical differences were found in DLCO % predicted between the 2 groups. A significant portion of the PPFE group required lung transplants (*n* = 3) compared to the non-PPFE group (*n* = 9), with a notable difference in the rates of transplantation between the groups (*p* = 0.0255), yet no significant disparity in the age at which transplants were performed (*p* = 0.51). See Table 1 for further details.

**Table 1:** A Baseline characteristics and other clinical data of patients with PPFE and non-PPFE

Characteristics	PPFE cohort (*n* = 6)	Non-PPFE cohort (*n* = 64)	*P* values
Age, yr			
Mean (SD)	56.5 ± 14.6	64.75 ± 10.7	0.229
Median (IQR)	61 (32–70)	67 (32–83)	
Female sex, no. (%)	5 (83%)	23 (36%)	0.0235
FVC, % predicted, no.	6	59	
Mean (SD)	47.8 ± 19.3	74.8 ± 21.0	0.017
DLCO, % predicted, no.	6	58	
Mean (SD)	43.5 ± 11.3	48.1 ± 23.7	0.426
Lung transplant, no. (%) Transplant age, yr	3 (50%)	9 (14%)	0.0255
Mean (SD)	51 ± 16.6	58.8 (11.8)	0.51

*Yr* years, *no* number, *FVC* force vital capacity, *DLCO* diffusing capacity of the lungs for carbon monoxide, *SD* standard deviation, *IQR* (interquartile range)

Of the 6 patients that were diagnosed with PPFE, five (83%) were found to have lymphocytes and granulocytes telomere length ≤ 10th percentile, and among these, four (80%) had a genetic variant in telomere-related genes. One in telomerase reverse transcriptase (*TERT*), one in telomerase RNA component (*TERC*), and two in regulator of telomere elongation helicase 1 (*RTEL1*). Two patients had personal and family features of Telomere Biology Disorders (TBD), including early graying of the hair, and/or hip dysplasia. A personal history of cancer was present in 67% of these patients. Three patients had end-stage lung disease and required lung transplantation. Lung biopsy confirmed the diagnosis of PPFE in five cases. Radiological imaging of PPFE was found in 4 cases. Additional baseline characteristics, mutation analysis and other clinical data are shown in Table 2.

**Table 2:** PPFE Individual baseline characteristics and other clinical data

	Gender	Age at diagnosis (years)	Time to diagnosis (years)	HRCT	Family history of lung fibrosis	Histology	FVC (%)	DLCO (%)	Telomere length lymphocytes (percentile)	Telomere length granulocytes (percentile)	Gene and variant	Other TBD features	Personal History of Cancer	TBD-related findings in the family	Lung transplant
1	F	64	4	PPFE	yes	PPFE	36	37	1–10	1–10	*RTEL1, VUS*(NM_001283009.2c.101A > G—p.(Gln34Arg))	No	Squamous cell carcinoma of the tongue and skin	Premature graying of hair	Yes
2	M	58	17	PPFE	Yes	PPFE	29	29	< 1	< 1	TERT, VUS* (NM_198253.3:c.2030G > A—p.(Gly677Asp))	Leukopenia Neutropenia	Squamous cell carcinoma of the left cheek	Hip dysplasia	Yes
3	F	70	3	Inconsistent with UIP	Yes	PPFE	57	75	1–10	< 1	*RTEL1, pathogenic* (NM_001283009.2c.2992C > T—p.(Arg974Ter))	No	No	No	No
4	F	32	4	PPFE	Yes	PPFE CHP	36	28	1–10	1–10	No exonic SNV	No	Breast cancer	No	Yes
5	F	47	1	PPFE	No	n/a	51	60	< 1	< 1	*TERC*, likely pathogenicNR_001566.1:n.54_57delAACT	No	No	No	No
6	F	68	6	Inconsistent with UIP	Yes	PPFE	52	58	> 10	> 10	*SFTPA2, likely pathogenic* (NM_001098668.4: c.532 G > A—p.(Val178Met))	No	Lung Adenocarcinoma	No	No

*HRCT* high-resolution computed tomography of the chest, *FVC* force vital capacity, *DLCO* diffusing capacity of the lungs for carbon monoxide, *TBD* telomere biology disorders, *F* female, *M* male, *VUS* variant of unknown significance, *SNV* single nucleotide variant, *UIP* usual interstitial pneumonia, *PPFE* pleuroparenchymal fibroelastosis, *CHP* chronic hypersensitivity pneumonitis

*Unpublished data from our group suggest that this variant affects telomerase activity

**Fig. 1:**
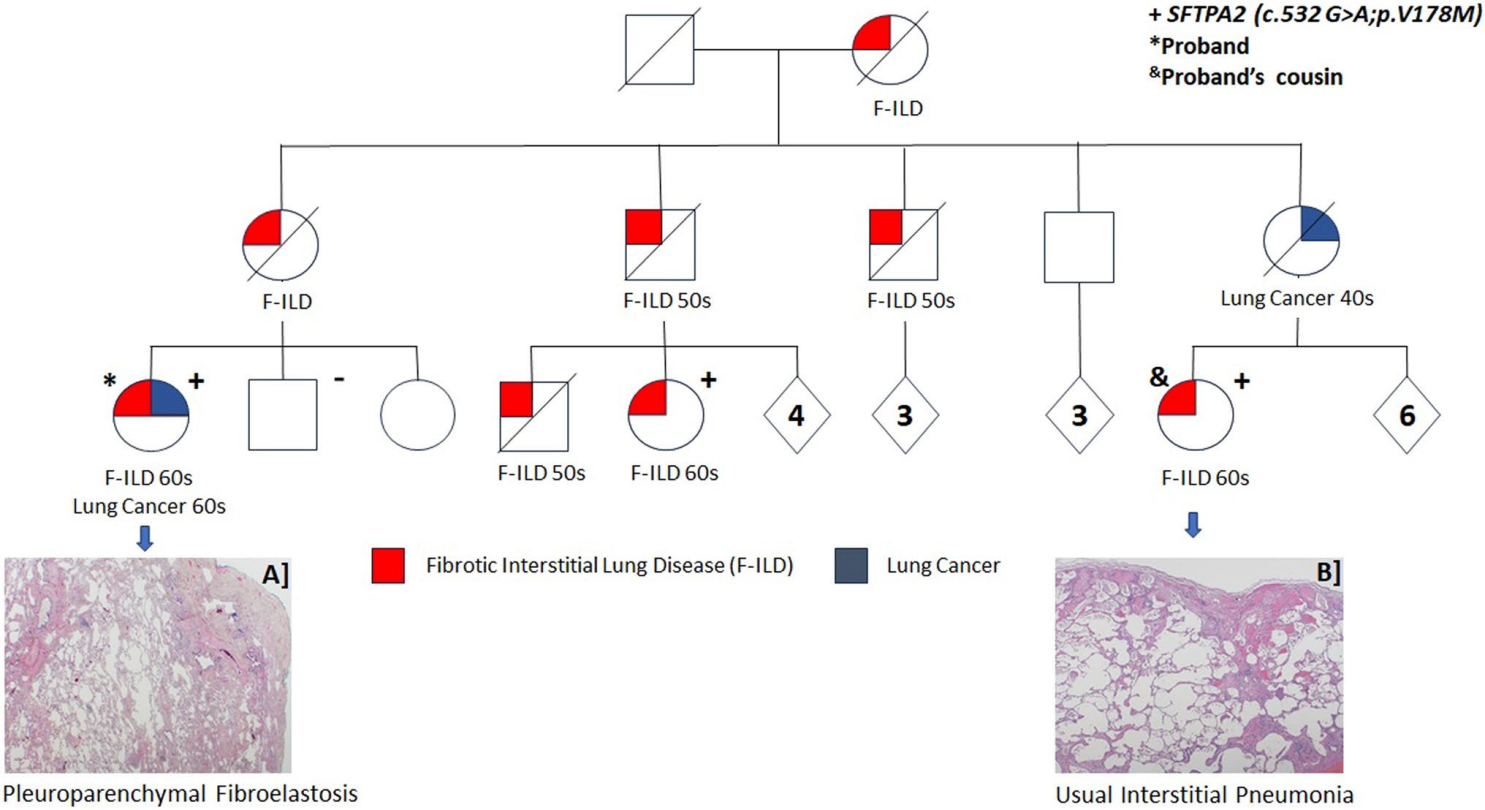
Pedigree of patient with a *SFTPA2* pathogenic variant (NM_001098668.4: c.532 G > A—p.(Val178Met)) and histological features. Multiple family members were affected with F-ILD and lung cancer, as shown. Pedigree also shows the 40X H&E lung tissue histology of proband (**A**) and proband’s cousin (**B**)

One patient (#6 of Table 2) had a heterozygous surfactant protein A2 (*SFTPA2*) variant (NM_001098668.4:c.532 G > A; p.(Val178Met)). This patient's maternal first cousin had the same variant, but interestingly, a lung biopsy showed UIP (pedigree and lung histology are shown in Fig. 1) with a radiographic pattern atypical for UIP.

## Discussion

FPF is estimated to account for at least 20% of patients with F-ILD [1]. A subset of FPF associates with short telomere length and genetic alterations in telomere-related (e.g., *TERT, TERC*, and *RTEL1*) and surfactant protein genes (e.g., *SFTPA1, SFTPA2, SFTPB,* and *SFTPC*). Conditions like IPF, chronic hypersensitivity pneumonitis (CHP), combined pulmonary fibrosis and emphysema, and PPFE are among some of the F-ILD associated with telomere biology disorders (TBD) and surfactant protein genetic alterations. These entities are indistinguishable from those with no genetic abnormalities. Furthermore, similar radiographic and histological findings may be the result of very different genetic alterations. Despite increasing recognition of the importance of genetic testing in F-ILD, its use is inconsistent. Increasing awareness and availability of clinical testing is therefore crucial.

In our study, we identified 6 patients (8%) with PPFE among a cohort of 70 individuals evaluated for FPF or early-onset lung fibrosis (*manuscript under preparation*), highlighting the prevalence of PPFE in this population. These PPFE patients were predominantly females, exhibited a lower FVC % predicted value, and had a higher likelihood of undergoing lung transplantation compared with the non-PPFE group. Although no significant differences were observed in the age at diagnosis, DLCO % predicted, or age at transplantation between the groups, the PPFE patients appeared to have more severe disease manifestations. However, due to the small sample size of the PPFE group, further research is necessary to validate these findings. The selection bias toward patients with a history of FPF or early lung fibrosis might have also influenced the observed severity of the disease.

Additionally, we found that five out of the six PPFE patients had evidence of TBD, either through shortened telomere length in peripheral lymphocytes or granulocytes (below the 10th percentile) or through the identification of pathogenic or likely pathogenic variants in telomere-associated genes. This is consistent with prior reports [9, 10]. Nunes et al. for example, described PPFE in 10.4% of patients with genetic-related pulmonary fibrosis [10]. Our findings underscore the variability in clinical presentation and the necessity for heightened clinical vigilance to identify TBD, as hallmark features like bone marrow failure, liver fibrosis, and early graying of hair may only manifest in a minority of affected individuals [11].

Interestingly, patient #6 in Table 2 who had a lung biopsy consistent with PPFE had a heterozygous pathogenic variant in the SFTPA2 gene. This genetic variant was also found in the patient's maternal first cousin. Although they shared the same genetic mutation, the cousin's lung biopsy revealed UIP, a diagnosis supported by lung histology. However, the radiographic pattern observed in the cousin was atypical for UIP, suggesting variability in the clinical presentation and radiological findings associated with the *SFTPA2* variant. This case highlights the genetic complexity and phenotypic diversity of interstitial lung diseases, particularly within familial contexts. The presence of the same genetic variant in relatives, manifesting in different pulmonary pathologies and radiographic patterns, underscores the importance of genetic testing and comprehensive clinical evaluation in the diagnosis and management of familial pulmonary fibrosis and related conditions. Pathogenic variants in *SFTPA1* and *SFTPA2* are also associated with lung malignancy, like in this case, with the risk of lung cancer in this group estimated to be as high as 36% as compared to 13% for individuals with IPF [12].

Genetic evaluation in cases of PPFE provides additional insights into the pathophysiology of the disease and can guide management since patients with TBD and surfactant protein abnormalities have a worse decline in FVC, reduced transplant-free survival, and poorer outcomes than those with normal telomere length or absence of genetic abnormalities [3, 4, 13, 14]. Avoidance of immunosuppressive agents and radiation exposure is also important, especially for those with TBD, as they are associated with worse outcomes [13, 14]. While the role of antifibrotics (nintedanib and pirfenidone) in TBD needs further investigation, newer data suggest that antifibrotics can be used safely and help reduce FVC declined in patients with TBD-associated lung fibrosis [15].

In summary, the presence of PPFE, especially in patients with premature lung fibrosis or familial disease, should prompt telomere length analysis and genetic evaluation to identify potentially pathogenic variants in telomere and surfactant protein-related genes. Genetic testing provides additional insight beyond radiological and histological findings and could avoid unnecessary testing and invasive procedures such as lung biopsy. It also has an impact on patients’ management and outcomes, as patients with TBD are sensitive to immunosuppression and radiotherapy and may need specific screening for other disease manifestations like liver cirrhosis and hematological disorders in patients with TBD and lung cancer in those with surfactant protein abnormalities [14] Identifying pathogenic genetic variants also leads to appropriate genetic counseling for patients and at-risk relatives.

## Data Availability

The data supporting this study's findings are available within the manuscript. Any additional data can be obtained from the corresponding author, [EMC], upon reasonable request.
